# Optimization and validation of ^18^F-DCFPyL PET radiomics-based machine learning models in intermediate- to high-risk primary prostate cancer

**DOI:** 10.1371/journal.pone.0293672

**Published:** 2023-11-09

**Authors:** Wietske I. Luining, Daniela E. Oprea-Lager, André N. Vis, Reindert J. A. van Moorselaar, Remco J. J. Knol, Maurits Wondergem, Ronald Boellaard, Matthijs C. F. Cysouw

**Affiliations:** 1 Department of Urology, Amsterdam University Medical Centers, Prostate Cancer Network Netherlands, Amsterdam, The Netherlands; 2 Department of Radiology & Nuclear Medicine, Amsterdam University Medical Centers, Cancer Center Amsterdam, Amsterdam, The Netherlands; 3 Department of Nuclear Medicine, Northwest Clinics, Alkmaar, The Netherlands; IRCCS Ospedale Policlinico San Martino, Genova, Italy, ITALY

## Abstract

**Introduction:**

Radiomics extracted from prostate-specific membrane antigen (PSMA)-PET modeled with machine learning (ML) may be used for prediction of disease risk. However, validation of previously proposed approaches is lacking. We aimed to optimize and validate ML models based on ^18^F-DCFPyL-PET radiomics for the prediction of lymph-node involvement (LNI), extracapsular extension (ECE), and postoperative Gleason score (GS) in primary prostate cancer (PCa) patients.

**Methods:**

Patients with intermediate- to high-risk PCa who underwent ^18^F-DCFPyL-PET/CT before radical prostatectomy with pelvic lymph-node dissection were evaluated. The training dataset included 72 patients, the internal validation dataset 24 patients, and the external validation dataset 27 patients. PSMA-avid intra-prostatic lesions were delineated semi-automatically on PET and 480 radiomics features were extracted. Conventional PET-metrics were derived for comparative analysis. Segmentation, preprocessing, and ML methods were optimized in repeated 5-fold cross-validation (CV) on the training dataset. The trained models were tested on the combined validation dataset. Combat harmonization was applied to external radiomics data. Model performance was assessed using the receiver-operating-characteristics curve (AUC).

**Results:**

The CV-AUCs in the training dataset were 0.88, 0.79 and 0.84 for LNI, ECE, and GS, respectively. In the combined validation dataset, the ML models could significantly predict GS with an AUC of 0.78 (*p*<0.05). However, validation AUCs for LNI and ECE prediction were not significant (0.57 and 0.63, respectively). Conventional PET metrics-based models had comparable AUCs for LNI (0.59, *p*>0.05) and ECE (0.66, *p*>0.05), but a lower AUC for GS (0.73, *p*<0.05). In general, Combat harmonization improved external validation AUCs (-0.03 to +0.18).

**Conclusion:**

In internal and external validation, ^18^F-DCFPyL-PET radiomics-based ML models predicted high postoperative GS but not LNI or ECE in intermediate- to high-risk PCa. Therefore, the clinical benefit seems to be limited. These results underline the need for external and/or multicenter validation of PET radiomics-based ML model analyses to assess their generalizability.

## Introduction

Risk stratification of primary prostate cancer (PCa) is important for determining prognosis and selecting optimal treatment strategies. Currently, this is based on clinical tumor stage (cT-stage), prostate-specific antigen (PSA) serum level, and the International Society of Urologic Pathology (ISUP) score in prostate biopsies [1]. These data are typically included in preoperative nomograms in patients without distant metastatic disease on conventional imaging (i.e., bone scintigraphy and computed tomography (CT)) and, more recently, prostate-specific membrane antigen (PSMA) positron emission tomography (PET) [2, 3]. These nomograms calculate the risk of lymph node involvement (LNI) and determine if extended pelvic lymph node dissection (ePLND) is indicated. However, ePLND remains the gold standard for determining lymph node status [1].

In recent years, modern imaging modalities, such as multiparametric magnetic resonance imaging (mpMRI) and PSMA-PET/CT, have been implemented in PCa care. Paralleled with the growth of artificial intelligence (AI) in medical imaging, this has triggered many investigations into AI-based image analysis for tumor staging and risk classification in primary PCa. One such approach is the use of machine learning (ML) models applied to radiomics data from prostate MRI or PSMA-PET/CT [4–7]. In radiomics, high-dimensional data is extracted from radiological images. ML algorithms can be trained to transform these radiomics data into clinically applicable predictions [8, 9].

In a previous analysis, we performed a machine learning-based analysis of ^18^F-DCFPyL (an ^18^F-labeled PSMA radioligand) PET/CT radiomics in patients with intermediate- to high-risk PCa scheduled for robot-assisted radical prostatectomy (RARP) with ePLND to predict LNI and high-risk tumor features, and observed excellent cross-validated predictive values [10]. However, this approach has not yet been validated to confirm its generalizability.

A known limitation of radiomics analyses is the often limited reproducibility caused by sensitivity to differences in imaging procedures (e.g., acquisition, reconstruction, segmentation) and factors related to radiomics calculation [9]. Therefore, in this study, we optimized and validated machine learning models based on ^18^F-DCFPyL PET radiomics, including Combat harmonization to compensate for possible center effects, in patients with intermediate- to high-risk primary PCa scheduled for RARP with ePLND [10].

## Patients and methods

### Patients

Patients with newly diagnosed, biopsy-proven intermediate- to high-risk PCa who underwent ^18^F-DCFPyL PET/CT imaging prior to RARP and ePLND were included. ePLND was performed if there were high-risk factors (i.e., serum PSA level >20 ng/mL, ISUP score ≥4, clinical or radiological tumor stage ≥3) or a nomogram-based risk of LNI of 8% or greater [1]. Patients underwent ^18^F-DCFPyL PET/CT at two centers in the Netherlands (Amsterdam University Medical Center (Amsterdam UMC) and the Northwest Clinics (NWZ) Alkmaar) between April 2017 and June 2020. Baseline clinical and pathology data were registered (i.e., PSA level, clinical tumor stage, biopsy ISUP score, and the percentage of positive prostate biopsies).

### Training and validation datasets

We used a training dataset (n = 72/76, excluding 4 patients with distant metastases from the previously published internal cross-validation (CV) [10]) from Amsterdam UMC to optimize methodologies and train the machine learning models. The models were applied to internal (n = 24) and external (n = 27) validation datasets from Amsterdam UMC and NWZ, respectively. This resulted in a combined validation dataset of 51 patients.

### Preoperative clinical and pathology data for baseline models

The radiomics-based machine learning models were compared to clinically used methods (henceforth referred to as baseline models) to assess their added value. The Memorial Sloan Kettering Cancer Center (MSKCC) nomogram was applied to calculate the risk of LNI and ECE and the corresponding AUCs [2]. The AUC of the biopsy Gleason score (GS) baseline model was calculated by using the biopsy GS as input.

### Postoperative pathology data

The surgical tissue specimens of the prostate and lymph nodes were examined according to the international guidelines of uropathologists for primary tumor and nodal staging [1]. The three selected reference outcomes for analysis were dichotomized, being postoperative GS (<8 vs. ≥8), presence of ECE (≤pT2b vs. ≥pT3a) and LNI (pN0 vs. pN1).

### Image acquisition and interpretation

PET input data (i.e., dose, calibration, injection, and scan times) were collected. At the Amsterdam UMC, patients were scanned on a time-of-flight PET/CT-scanner (Ingenuity or Vereos, Philips Healthcare) with European Association of Nuclear Medicine (EANM) Research Ltd. (EARL) accreditation [11]. Whole-body PET-images were obtained from mid-thigh to skull base at 4 minutes per bed position at 119 minutes (interquartile range (IQR) 116–125) post-injection (p.i.) of a median dose of 308 MBq (IQR 293–318) ^18^F-DCFPyL. Data were reconstructed using the vendor-provided iterative reconstruction algorithm using 3 iterations and 33 subsets with 4 mm isotropic voxels (EARL-compliant). PET-images were combined with a non-contrast-enhanced low-dose CT-scan (30–110 mAs at 120 kV).

At the NWZ, scans were performed on a Biograph-16 TruePoint PET/CT-scanner (Siemens Healthcare). At 114 minutes (IQR 110–122) p.i. of a median activity of 302 MBq (IQR 270–338, depending on body mass) ^18^F-DCFPyL, whole-body PET from mid-thigh to skull base was acquired. The acquisition time was 5 minutes per bed position. Images were reconstructed using iterative ordered-subset expectation maximization reconstruction (4 iterations, 16 subsets) with 2.67x2.67x4.0 mm voxels. PET-scans were combined with a contrast-enhanced CT-scan (110 mAs at 130 kV).

PET-images were corrected for scatter, decay, randoms, and attenuation. In addition to the originally reconstructed images, the Lucy-Richardson iterative deconvolution was applied for partial volume correction (PVC) [12].

### Tumor segmentation

The latest version of the ACCURATE tool was used for tumor segmentation on PET-images [13]. A mask was manually drawn around the PSMA-avid prostatic lesion on PET to prevent the inclusion of activity from other structures, such as the urinary bladder or rectum. The masks were reviewed by a second observer. If necessary, the masks were jointly adjusted. Subsequently, the tumor was delineated semi-automatically using a region-growing isocontour corresponding to 50%, 55%, 60%, 65%, and 70% of the peak Standardized Uptake Value (SUV) with or without correction for local background uptake. The original and PVC images were delineated separately.

### Radiomics feature extraction

Radiomics features were calculated from the segmented region using the Image Biomarker Standardization Initiative (IBSI)-compliant RaCat software [14, 15]. Voxels were resampled to 2 mm isotropic voxels using tri-linear interpolation with aligned edges [16, 17]. Voxels were scaled to the SUV. Before calculating textural features, discretization was applied with a fixed bin width of 0.25 SUV units. A total of 480 radiomics features on texture (n = 408), intensity (n = 50) and morphology (n = 22) were extracted per patient. Additionally, conventional PET metrics (e.g., SUV values, volume, and total PSMA uptake) were derived for comparative analysis.

### Machine learning

Random Forest (RF) and Logistic Regression (LR) were used as machine learning classifiers. For data normalization, we applied i) z-score standardization and ii) Yeo-Johnson transformation [18]. Dimension reduction was performed using i) Principal Component Analysis, ii) Recursive Feature Elimination, iii) univariate feature selection, and iv) the Least Absolute Shrinkage and Selection Operator [19]. The Synthetic Minority Oversampling Technique (SMOTE) was applied to correct data imbalance [20]. To mitigate the center effect, Combat harmonization (using the *neuroCombat* implementation) was applied to external radiomics data, using the training data as reference dataset [21, 22]. Also, the impact of filtering features (based on linear correlations) before dimension reduction on was evaluated. Lastly, the influence of adding clinical parameters (initial PSA and biopsy ISUP score) to the data after radiomics feature selection was evaluated.

The optimal combination of tumor segmentation settings, use of PVC, and machine learning methods (data normalization, dimension reduction, oversampling) was selected using 10-times repeated 5-fold CV on the training dataset. Model hyperparameters were optimized in nested cross-validation. The models with optimal machine learning configurations were then trained on the entire training dataset and tested on the validation datasets (settings in Table 1).

**Table 1:** The optimal machine learning configuration based on the cross-validated AUCs of the training dataset.

Classifier	Isocontour threshold*	Background correction	PVC	Data normalization	Oversampling	Dimension reduction
LNI						
RF	65%	On	Yes	Powertransformer	None	LASSO
LR	65%	On	Yes	Powertransformer	SMOTE	Univariate
ECE						
RF	60%	On	No	Z-score	SMOTE	LASSO
LR	60%	On	No	Z-score	None	LASSO
GS						
RF	70%	Off	Yes	Powertransformer	SMOTE	RFE
LR	60%	On	Yes	Z-score	None	LASSO

AUC = area under the curve; LNI = lymph node involvement; ECE = extracapsular extension; GS = Gleason score; RF = random forest; LR = logistic regression; VOI = volume of interest; PVC = partial volume correction; RFE = Recursive Feature Elimination; LASSO = Least Absolute Shrinkage and Selection Operator, SMOTE = synthetic minority over-sampling technique.

*treshold defined as percentage of SUVpeak

### Statistical analysis

The area under the curve (AUC) of the receiver operator characteristic (ROC) curve, sensitivity and specificity was calculated to assess model performance. In CV, the mean AUC (CV-AUC) was calculated. In each CV iteration, the probability threshold at the Youden index was determined. The mean probability threshold was then used to calculate sensitivity and specificity in the validation datasets. The DeLong test was performed to compare the AUC of different models applied to the validation data [23]. Machine learning analyses were executed in Python 3.7 using the SciKit package [24]. Statistical analysis was performed using SPSS version 28 and GraphPad Prism version 9. Statistical significance was considered at *p*<0.05.

## Results

### Patients and clinical characteristics

Pre- and postoperative clinicopathological characteristics are listed in Tables 2 and 3, respectively. The distribution of intermediate- and high-risk disease according to European Urology (EAU) guidelines for the training dataset was 38.9% (28/72) versus 61.1% (44/72), and 27.5% (14/51) versus 72.5% (37/51) in the combined validation dataset, respectively [1].

**Table 2:** Preoperative characteristics of all included patients per dataset.

	Training	Internal validation	External validation
Patients, no	72	24	27
Age at scan, yr, median (IQR)	67 (61–70)	70 (62–74)	68 (59–70)
PSA at PET, ng/mL, median (IQR)	11.0 (7.3–21.0)	9.4 (4.4–17.9)	12.2 (9.1–23.0)
Positive biopsies, %, median (IQR)	50.0 (33.3–71.4)	60.0 (42.5–85.0)	62.5 (50.0–75.0)
Biopsy ISUP grade group, no (%)*			
ISUP 1	4 (5.6)	0 (0.0)	1 (3.7)
ISUP 2	20 (27.8)	4 (16.7)	10 (37.0)
ISUP 3	16 (22.2)	9 (37.5)	4 (14.8)
ISUP 4	21 (29.2)	7 (29.2)	7 (25.9)
ISUP 5	11 (15.3)	4 (16.7)	5 (18.5)
Clinical T stage, no (%)			
T1c	25 (34.7)	5 (20.8)	15 (55.6)
T2a/b	36 (50.0)	11 (45.9)	9 (33.3)
T2c	9 (12.5)	5 (20.8)	0 (0.0)
T3	2 (2.8)	3 (12.5)	3 (11.1)

IQR = interquartile range; PSA = prostate-specific antigen; PET = positron emission tomography; ISUP = International Society of Urological Pathology.

* ISUP Definition

ISUP 1 = Gleason score 3 + 3 = 6

ISUP 2 = Gleason score 3 + 4 = 7

ISUP 3 = Gleason score 4 + 3 = 7

ISUP 4 = Gleason score 4 + 4 = 8/ Gleason score 3 + 5 = 8 / Gleason score 5 + 3 = 8

ISUP 5 = Gleason score 4 + 5 = 9/ Gleason score 5 + 4 = 9/ Gleason score 5 + 5 = 10

**Table 3:** Final histopathological results of all included patients who underwent radical prostatectomy and extended pelvic lymph node dissection per dataset.

	Training dataset	Internal validation	External validation
Pathological T stage, no (%)			
pT2	35 (49.3)	11 (45.8)	12 (44.4)
pT3a	25 (35.2)	8 (33.3)	11 (40.7)
pT3b	10 (14.1)	5 (20.8)	4 (14.8)
pT4	1 (1.4)	0 (0.0)	0 (0.0)
Missing	1		
Pathological ISUP grade group, no (%)*			
ISUP 1	1 (1.4)	0 (0.0)	0 (0.0)
ISUP 2	27 (38.0)	5 (20.8)	12 (44.4)
ISUP 3	24 (33.8)	12 (50.0)	8 (29.6)
ISUP 4	5 (7.0)	1 (4.2)	2 (7.4)
ISUP 5	14 (19.7)	6 (25.0)	5 (18.5)
Missing	1		
Surgical margin status, no (%)			
R0	43 (60.6)	16 (66.7)	15 (55.6)
R1	28 (39.4)	8 (33.3)	12 (44.4)
Missing	1		
Pathological N stage, no (%)			
N0	62 (86.1)	18 (75.0)	22 (81.5)
N1	10 (13.9)	6 (25.0)	5 (18.5)

IQR = interquartile range; PSA = prostate-specific antigen; PET = positron emission tomography; ISUP = International Society of Urological Pathology.

* ISUP Definition

ISUP 1 = Gleason score 3 + 3 = 6

ISUP 2 = Gleason score 3 + 4 = 7

ISUP 3 = Gleason score 4 + 3 = 7

ISUP 4 = Gleason score 4 + 4 = 8/ Gleason score 3 + 5 = 8/ Gleason score 5 + 3 = 8

ISUP 5 = Gleason score 4 + 5 = 9/ Gleason score 5 + 4 = 9/ Gleason score 5 + 5 = 10

### Model validation

**Fig 1:**
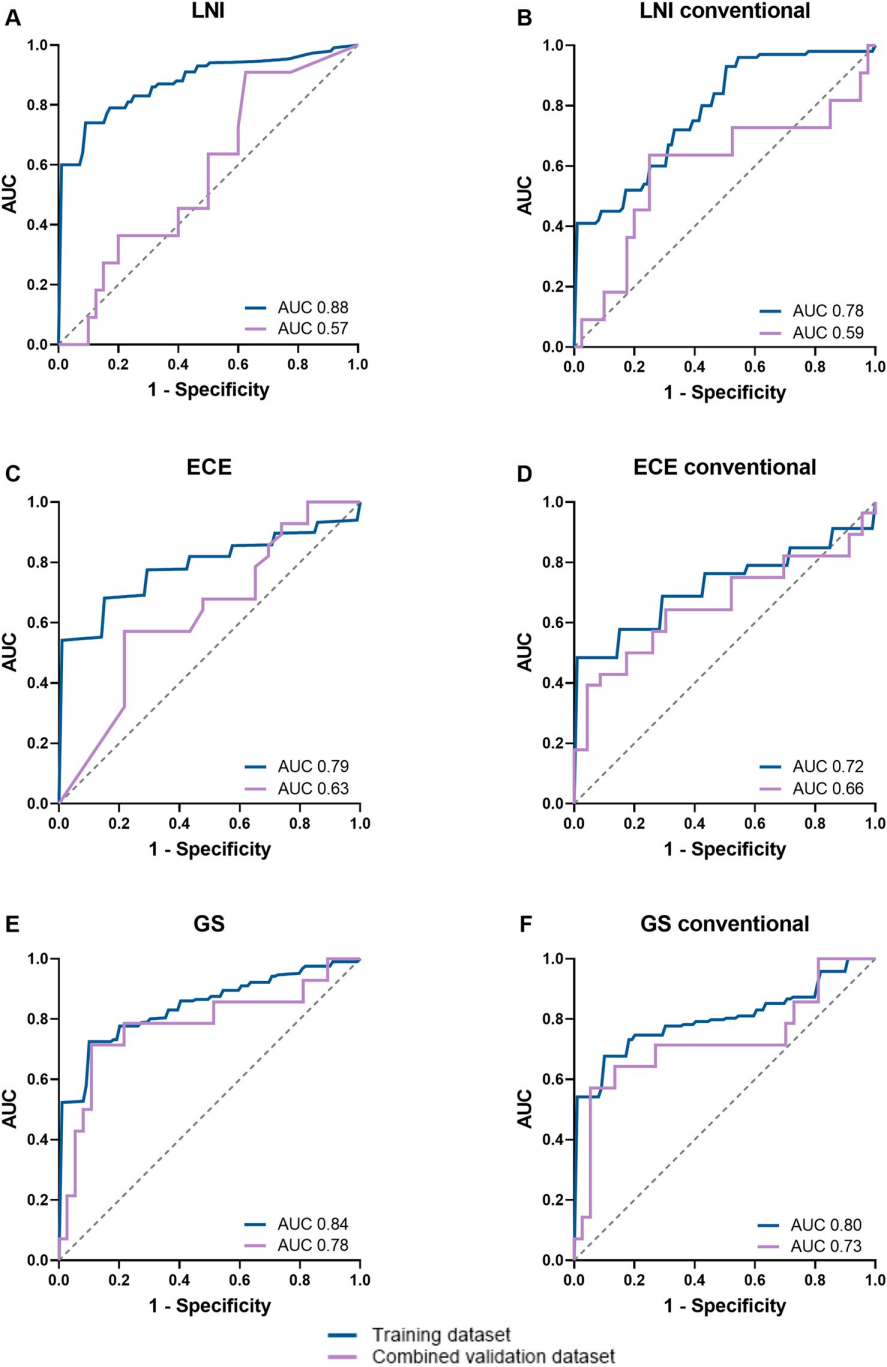
ROC-curves of the machine learning models using radiomics features or conventional PET-metrics for predicting lymph node involvement (LNI; A,B), extracapsular extension (ECE; C,D), and Gleason score (GS; E,F) in the training dataset or combined validation cohort. Training dataset AUC is the mean cross-validation AUC.

Highest CV-AUCs in the training dataset were 0.88 for LNI, 0.79 for ECE, and 0.84 for GS, respectively (all using the Random Forest classifier; see Table 1 for configurations). The radiomics models outperformed the conventional metrics models in CV (LNI 0.78, ECE 0.72, and GS 0.80). Compared to the CV-AUCs, in the combined validation dataset the radiomics-based machine learning models yielded lower non-significant AUCs for LNI (0.57) and ECE (0.63), but a significant AUC of 0.78 for GS (*p*<0.05). In the combined validation dataset, the models based on conventional PET metrics had similar AUCs for LNI (0.59, *p*>0.05) and ECE (0.66, *p*>0.05), and a lower, but significant AUC for GS (0.73, *p*<0.05). No significant differences were found between the radiomics- and conventional PET metrics-based AUCs of the combined validation dataset. The AUCs, sensitivity and specificity of the radiomics-based machine learning models are listed in Table 4. The ROC-curves for these analyses are given in Fig 1. The AUCs of the different machine learning classifiers with/without Combat harmonization or the addition of clinical parameters for predicting LNI, ECE, and GS are summarized in S1 Table.

**Table 4:** The AUC, sensitivity and specificity of the radiomics-based models per outcome.

	AUC	Sensitivity	Specificity
Training dataset (±SD)*			
LNI	0.88 ± 0.16	0.93 ± 0.20	0.88 ± 0.15
ECE	0.79 ± 0.12	0.71 ± 0.18	0.91 ± 0.11
GS	0.84 ± 0.13	0.86 ± 0.17	0.86 ± 0.15
Validation dataset (95% CI)			
LNI	0.57 (0.40–0.75)	0.18 (0.032–0.48)	0.88 (0.74–0.95)
ECE	0.63 (0.47–0.79)	0.57 (0.39–0.73)	0.78 (0.58–0.90)
GS	0.78 (0.61–0.95)	0.69 (0.42–0.87)	0.84 (0.69–0.92)

AUC = area under the curve; LNI = lymph node involvement; ECE = extracapsular extension; GS = Gleason score; CI = confidence interval; SD = standard deviation.

*cross-validated AUC

### Impact of combat harmonization

**Fig 2:**
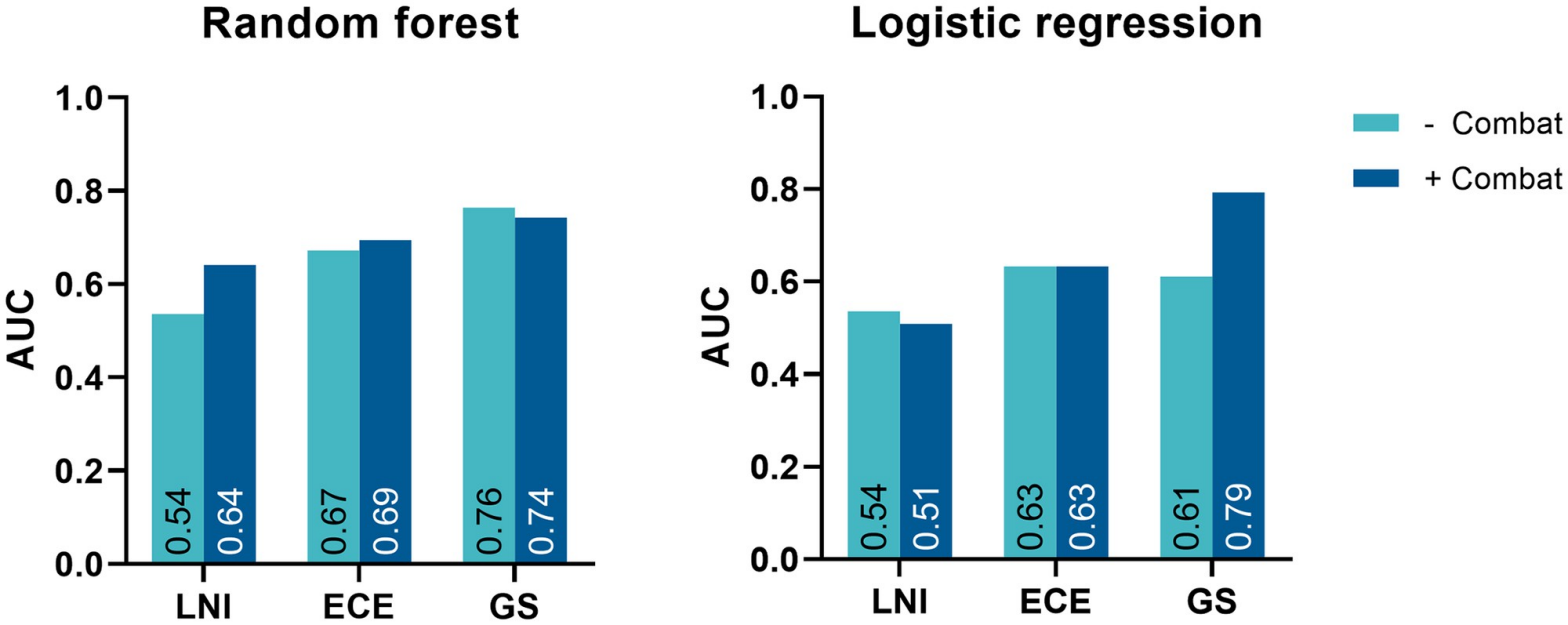
The effect of Combat harmonization in the external validation dataset on model performance per machine learning classifier for lymph node involvement (LNI), extracapsular extension (ECE), and Gleason score (GS) prediction.

The effect of Combat harmonization on the performance of the radiomics-based machine learning models in the external validation dataset for LNI, ECE, and GS predictions is illustrated in Fig 2. In general, Combat harmonization improved the external validation AUCs with a range of -0.03 to +0.18.

### Impact of adding clinical data

**Fig 3:**
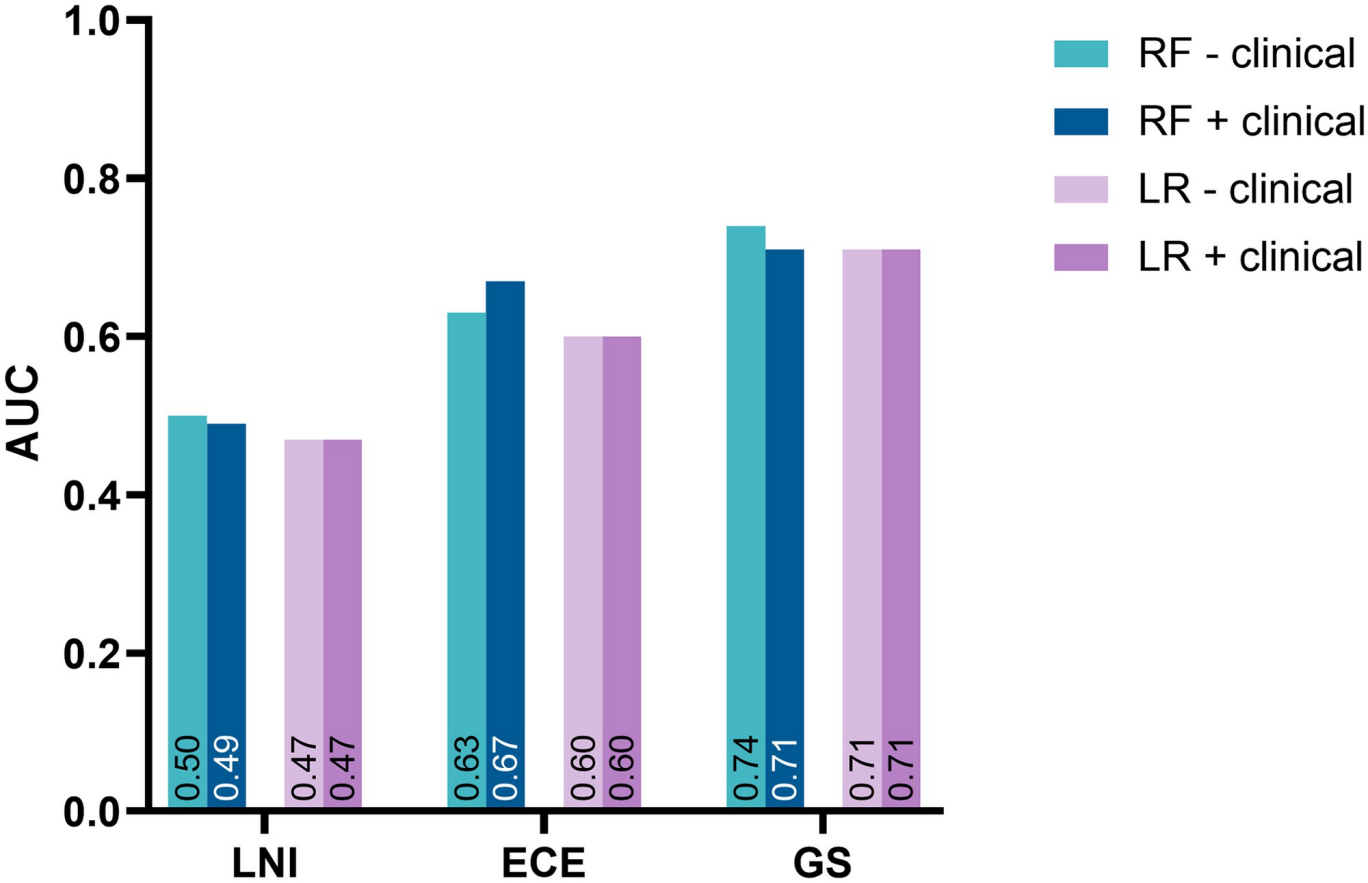
The effect of adding clinical parameters to radiomics data on model performance for the combined validation dataset.

Adding clinical parameters (initial PSA and biopsy ISUP score) to the radiomics data after feature selection did not improve model performance for LNI, ECE, or GS prediction for the combined validation dataset (Fig 3).

### Comparison with baseline models

**Fig 4:**
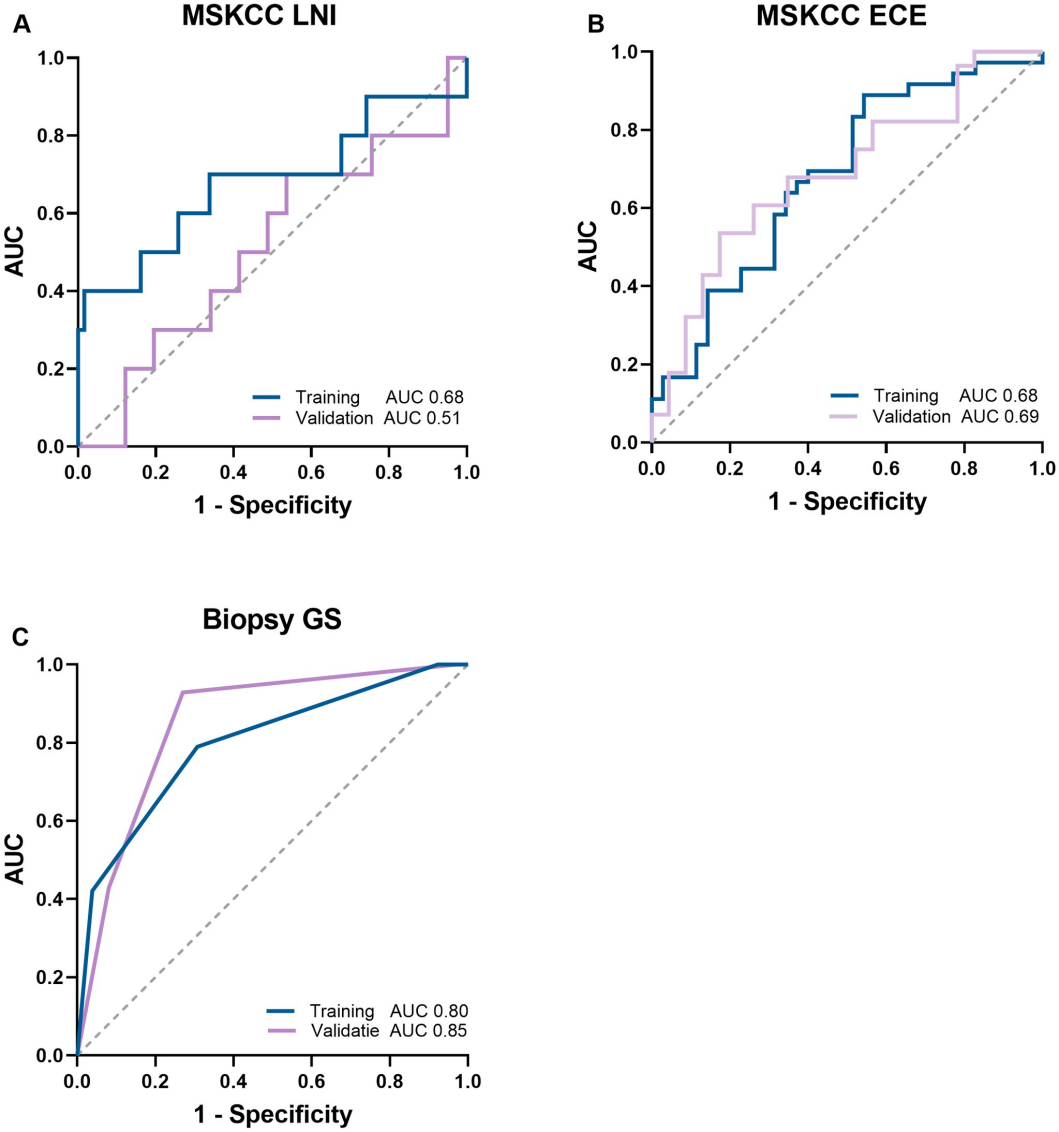
ROC-curves of the baseline models in the training dataset and the combined validation dataset. The pre-radical prostatectomy MSKCC-nomogram for lymph node involvement (LNI; A) and extracapsular extension (ECE; B) prediction and the biopsy baseline model for postoperative Gleason score (GS; C) prediction.

The AUC of the MSKCC-nomogram for predicting LNI was 0.68 (*p*>0.05) in the training dataset and 0.51 (*p*>0.05) in the combined validation dataset. The AUC of the MSKCC-nomogram for predicting ECE was 0.68 (*p*<0.05) in the training dataset and 0.69 (*p*<0.05) in the combined validation dataset. The AUC of the biopsy GS baseline model for predicting final GS was 0.80 (*p*<0.05) in the training and 0.85 (*p*<0.05) in the combined validation dataset. The ROC-curves of the baseline models are illustrated in Fig 4.

## Discussion

In this study, we optimized and validated the performance of a previously proposed machine-learning approach using ^18^F-DCFPyL PET radiomics for predicting high-risk tumor features in patients with intermediate- to high-risk PCa in a multicenter dataset. In this validation, we found that ^18^F-DCFPyL PET radiomics could predict high postoperative GS but not LNI and ECE. Therefore, the clinical benefit seems to be limited. Still, this implies that radiomics-based machine learning models could enhance preoperative GS determination in clinical practice and potentially help clinicians in their daily decision-making process. The discrepancy between positive results from internal cross-validation and negative results from multicenter validation emphasizes the importance of such validation studies for radiomics analyses.

Newly diagnosed patients with PCa are typically stratified into three risk groups (i.e., low-, intermediate-, and high-risk PCa) based on PSA level, clinical T-stage, and biopsy GS [1]. Accurate prediction of the GS is essential for treatment guidance, to avoid over- or undertreatment. Not all patients undergo radical prostatectomy, so GS obtained with biopsy is usually used in daily practice regardless of the knowledge of discordance between biopsy and prostatectomy GS [25, 26]. However, since the introduction of targeted prostate biopsies, pathological upgrading at prostatectomy has decreased significantly. A recent systematic review and meta-analysis by Goel et al. evaluated the concordance between systematic or MRI-targeted prostate biopsies and prostatectomy GS. They found that pathologic upgrading at prostatectomy was significantly less frequent with targeted biopsy (23.3%) compared to systemic biopsy alone (42.7%), without significant differences in pathological downgrading between the two biopsy techniques [26]. Our present study, using radiomics-based models, could predict postoperative GS with an AUC of 0.78, and the AUC of our baseline model was 0.85. This high AUC of our baseline model might be explained by the improved accuracy of (target) prostate biopsies to determine the GS correctly. However, biopsies are subsamples of the prostate, making it subject to sampling errors, and minor complications (i.e., bleeding or infection) are frequent. Therefore, radiomics-based machine learning models could be a helpful non-invasive tool to assist in GS determination in patients with primary PCa.

Several studies have assessed the value of PSMA-PET radiomics to predict GS in PCa [27–31]. However, multicenter validation is often lacking [32]. Zamboglou et al. reported similar results AUC for GS discrimination (GS 7 vs. ≥8) with ^68^Ga-PSMA-PET radiomics in their internal validation cohort [31]. Papp et al. found that radiomics combined with machine learning could discriminate between low- and high-risk PCa lesions on ^68^Ga-PSMA-PET/MR [28]. The use of ^68^Ga-PSMA-PET/MR radiomics was also investigated by Solari et al., who reported an AUC of 0.75 for predicting postsurgical GS with their PET radiomics model. Their method differed from ours in that they segmented the whole prostate gland and trained their model to predict three instead of two GS categories (GS <8, 8, and >8) [29]. Recently, Aksu et al. evaluated whether ^68^Ga-PSMA-PET/CT radiomics (without machine learning) could predict GS ≥8 and found an AUC of 0.90 [27]. Using ^18^F-1007-PSMA-PET radiomics, Yao et al. found a similar predictive performance for GS characterization (training AUC 0.82; testing AUC 0.80) [30]. In summary, our results are consistent with previous research, suggesting that PSMA-PET radiomics could be of added value for the preoperative prediction of postoperative GS.

In our training dataset, we found high cross-validation prediction scores for each outcome, whereas in the validation dataset, the radiomics-based models could not predict LNI and ECE (AUCs of 0.57 and 0.63). The same applied to the models using conventional PET metrics as input, implying that the issue is not merely related to the robustness of texture-based radiomics features. Also, two baseline models (MSKCC nomogram predictions for LNI and ECE) reached higher or equal AUCs in the training dataset compared to the validation cohort. Taken together, this may indicate that the low prediction AUCs in the (combined internal and external) validation datasets are due to inherent characteristics of the validation dataset instead of merely overfitted or biased models. In contrast, the performance of the biopsy baseline models to predict postoperative GS reached higher AUCs in the validation dataset compared to the training dataset. This prediction is based only on the biopsy GS, which relies on various factors such as the biopsy technique (systematic versus targeted), the number of biopsies taken, and the physician’s experience performing the procedure.

As is well known, radiomics features (especially those based on texture) are sensitive to variations in PET systems, image acquisition protocols, reconstruction settings, post-processing, and segmentation methods. As the purpose of this study was a multicenter validation, the validation dataset consisted of patients from two different institutions with differences in PET systems, reconstruction software, and imaging protocols. A radiomics-based machine learning model trained with data from one center may not apply directly to data from another. Therefore, to minimize the influence of a center effect, Combat harmonization was applied to harmonize the radiomics features. Indeed, this improved our validation AUCs of the external dataset. To date, the use of Combat harmonization in PCa radiomics analyses is limited. However, the Combat harmonization method has been used in several contexts, and its efficacy in PET imaging has been demonstrated [22, 33–35].

Our study is not devoid of limitations. First, most patients had high-risk disease according to the EAU guidelines, and our results should also be validated in patient cohorts with lower-risk disease. Secondly, we only selected patients who had undergone radical prostatectomy as we chose postoperative GS as one of the reference outcomes. However, patients undergoing radiotherapy instead of radical prostatectomy are not expected to differ much from the current population. Thirdly, the anatomical locations of the prostate tumors delineated on PET were not directly compared to the prostatectomy specimen. Potentially, less PSMA-avid lesions on PET may be missed in, for example, multifocal tumors. In clinical practice, however, anatomical information from the prostatectomy specimen is, logically, not available at pre-operative imaging. We chose to delineate the most PSMA-avid foci within the prostate based on semi-automatic lesion segmentation. This could impact the radiomics feature values and, consequently, our results. Still, our results are in line with other data where whole-prostate radiomics were derived [29]. Also, semi-automatic segmentation analyses suffer less from observer variability than manual segmentations [36]. In future prospective research, additional information on disease recurrence during follow-up may help evaluate the utility of the machine-learning algorithm in treating patients with PCa.

## Conclusion

The ^18^F-DCFPyL radiomics-based machine learning models could predict high postoperative GS but not LNI or ECE. Still, these results are in line with the baseline models. This implies that radiomics-based machine learning models could enhance preoperative GS determination in clinical practice and potentially help clinicians in their daily decision-making process. Furthermore, these results underline the need for external and/or multicenter validation of PET radiomics-based machine learning model analyses to investigate their reproducibility and clinical applicability.

## Supporting information

S1 Table The different AUCs for the prediction of lymph node involvement (LNI), extracapsular extension (ECE), and Gleason score (GS) per dataset with or without the addition of clinical features, omission of correlated radiomics features, and application of Combat harmonization. AUC = area under the curve; RF = random forest; LR = logistic regression; LNI = lymph-node involvement; GS = Gleason score; ECE = extracapsular extension; SD = standard deviation; int. = internal; ext. = external; val. = validation; RFE = Recursive Feature Elimination; LASSO = Least Absolute Shrinkage and Selection Operator. *The number of radiomics features remaining after dimension reduction.
(DOCX)

S2 Table Selected features after dimension reduction for the prediction of lymph node involvement (LNI), extracapsular extension (ECE), and Gleason score (GS). Derived from a Random Forest and Logistic Regression using different machine-learning configurations (Table 1).
(DOCX)

## References

[1] EAU Guidelines. Edn. presented at the EAU Annual Congress Amsterdam 2022. ISBN 978-94-92671-16-5. Website. https://uroweb.org/ 2022.

[2] Memorial Sloan Kettering Cancer Centre. Prostate cancer nomograms: pre-radical prostatectomy. 2020.

[3] Gandaglia G, Fossati N, Zaffuto E, Bandini M, Dell’Oglio P, Bravi CA, et al. Development and Internal Validation of a Novel Model to Identify the Candidates for Extended Pelvic Lymph Node Dissection in Prostate Cancer. Eur Urol. 2017;72:632–40. doi: 10.1016/j.eururo.2017.03.049 28412062

[4] Ghezzo S, Bezzi C, Presotto L, Mapelli P, Bettinardi V, Savi A, et al. State of the art of radiomic analysis in the clinical management of prostate cancer: A systematic review. Critical Reviews in Oncology/Hematology. 2022;169:103544. doi: 10.1016/j.critrevonc.2021.103544 34801699

[5] Guglielmo P, Marturano F, Bettinelli A, Gregianin M, Paiusco M, Evangelista L. Additional Value of PET Radiomic Features for the Initial Staging of Prostate Cancer: A Systematic Review from the Literature. Cancers; 2021. doi: 10.3390/cancers13236026 34885135 PMC8657371

[6] Ma K, Harmon SA, Klyuzhin IS, Rahmim A, Turkbey B. Clinical Application of Artificial Intelligence in Positron Emission Tomography: Imaging of Prostate Cancer. PET Clinics. 2022;17:137–43. doi: 10.1016/j.cpet.2021.09.002 34809863

[7] Spohn SKB, Bettermann AS, Bamberg F, Benndorf M, Mix M, Nicolay NH, et al. Radiomics in prostate cancer imaging for a personalized treatment approach—current aspects of methodology and a systematic review on validated studies. Theranostics. 2021;11:8027–42. doi: 10.7150/thno.61207 34335978 PMC8315055

[8] Lambin P, Rios-Velazquez E, Leijenaar R, Carvalho S, Van Stiphout RG, Granton P, et al. Radiomics: extracting more information from medical images using advanced feature analysis. European journal of cancer. 2012;48:441–6. doi: 10.1016/j.ejca.2011.11.036 22257792 PMC4533986

[9] Zwanenburg A. Radiomics in nuclear medicine: robustness, reproducibility, standardization, and how to avoid data analysis traps and replication crisis. Eur J Nucl Med Mol I. 2019;46:2638–55. doi: 10.1007/s00259-019-04391-8 31240330

[10] Cysouw MCF, Jansen BHE, van de Brug T, Oprea-Lager DE, Pfaehler E, de Vries BM, et al. Machine learning-based analysis of [(18)F]DCFPyL PET radiomics for risk stratification in primary prostate cancer. Eur J Nucl Med Mol Imaging. 2020. doi: 10.1007/s00259-020-04971-z 32737518 PMC7835295

[11] Boellaard R, Delgado-Bolton R, Oyen WJG, Giammarile F, Tatsch K, Eschner W, et al. FDG PET/CT: EANM procedure guidelines for tumour imaging: version 2.0. Eur J Nucl Med Mol I. 2015;42:328–54. doi: 10.1007/s00259-014-2961-x 25452219 PMC4315529

[12] Cysouw MCF, Kramer GM, Hoekstra OS, Frings V, de Langen AJ, Smit EF, et al. Accuracy and Precision of Partial-Volume Correction in Oncological PET/CT Studies. Journal of Nuclear Medicine. 2016;57:1642. doi: 10.2967/jnumed.116.173831 27230933

[13] Boellaard R. Quantitative oncology molecular analysis suite: ACCURATE. Soc Nuclear Med; 2018.

[14] Pfaehler E, Zwanenburg A, de Jong JR, Boellaard R. RaCaT: An open source and easy to use radiomics calculator tool. PLoS One. 2019;14:e0212223. doi: 10.1371/journal.pone.0212223 30785937 PMC6382170

[15] Zwanenburg A, Leger S, Vallières M, Löck S. Image biomarker standardisation initiative. arXiv preprint arXiv:161207003. 2016.

[16] Hatt M, Tixier F, Pierce L, Kinahan PE, Le Rest CC, Visvikis D. Characterization of PET/CT images using texture analysis: the past, the present… any future? Eur J Nucl Med Mol I. 2017;44:151–65. 10.1007/s00259-016-3427-0 PMC5283691 27271051

[17] Pfaehler E, van Sluis J, Merema BB, van Ooijen P, Berendsen RC, van Velden FH, et al. Experimental multicenter and multivendor evaluation of PET radiomic features performance using 3D printed phantom inserts. Journal of Nuclear Medicine. 2019. 10.2967/jnumed.119.229724 PMC7067530 31420497

[18] Yeo IK, Johnson RA. A new family of power transformations to improve normality or symmetry. Biometrika. 2000;87:954–9. doi: 10.1093/biomet/87.4.954

[19] Tibshirani R. Regression shrinkage and selection via the lasso. Journal of the Royal Statistical Society: Series B (Methodological). 1996;58:267–88.

[20] Chawla NV, Bowyer KW, Hall LO, Kegelmeyer WP. SMOTE: synthetic minority over-sampling technique. Journal of artificial intelligence research. 2002;16:321–57.

[21] Fortin J-P, Cullen N, Sheline YI, Taylor WD, Aselcioglu I, Cook PA, et al. Harmonization of cortical thickness measurements across scanners and sites. NeuroImage. 2018;167:104–20. doi: 10.1016/j.neuroimage.2017.11.024 29155184 PMC5845848

[22] Orlhac F, Eertink JJ, Cottereau A-S, Zijlstra JM, Thieblemont C, Meignan M, et al. A Guide to ComBat Harmonization of Imaging Biomarkers in Multicenter Studies. Journal of Nuclear Medicine. 2022;63:172. doi: 10.2967/jnumed.121.262464 34531263 PMC8805779

[23] DeLong ER, DeLong DM, Clarke-Pearson DL. Comparing the areas under two or more correlated receiver operating characteristic curves: a nonparametric approach. Biometrics. 1988;44:837–45. 3203132

[24] Pedregosa F, Varoquaux G, Gramfort A, Michel V, Thirion B, Grisel O, et al. Scikit-learn: Machine learning in Python. the Journal of machine Learning research. 2011;12:2825–30.

[25] Cohen MS, Hanley RS, Kurteva T, Ruthazer R, Silverman ML, Sorcini A, et al. Comparing the Gleason prostate biopsy and Gleason prostatectomy grading system: the Lahey Clinic Medical Center experience and an international meta-analysis. Eur Urol. 2008;54:371–81. doi: 10.1016/j.eururo.2008.03.049 18395322

[26] Goel S, Shoag JE, Gross MD, Al Hussein Al Awamlh B, Robinson B, Khani F, et al. Concordance Between Biopsy and Radical Prostatectomy Pathology in the Era of Targeted Biopsy: A Systematic Review and Meta-analysis. Eur Urol Oncol. 2020;3:10–20. doi: 10.1016/j.euo.2019.08.001 31492650

[27] Aksu A, Vural Topuz Ö, Yılmaz G, Çapa Kaya G, Yılmaz B. Dual time point imaging of staging PSMA PET/CT quantification; spread and radiomic analyses. Ann Nucl Med. 2022;36:310–8. doi: 10.1007/s12149-021-01705-5 34988888

[28] Papp L, Spielvogel CP, Grubmuller B, Grahovac M, Krajnc D, Ecsedi B, et al. Supervised machine learning enables non-invasive lesion characterization in primary prostate cancer with [(68)Ga]Ga-PSMA-11 PET/MRI. Eur J Nucl Med Mol Imaging. 2020. doi: 10.1007/s00259-020-05140-y 33341915 PMC8113201

[29] Solari EL, Gafita A, Schachoff S, Bogdanović B, Villagrán Asiares A, Amiel T, et al. The added value of PSMA PET/MR radiomics for prostate cancer staging. Eur J Nucl Med Mol Imaging. 2022;49:527–38. doi: 10.1007/s00259-021-05430-z 34255130 PMC8803696

[30] Yao F, Bian S, Zhu D, Yuan Y, Pan K, Pan Z, et al. Machine learning-based radiomics for multiple primary prostate cancer biological characteristics prediction with 18F-PSMA-1007 PET: comparison among different volume segmentation thresholds. La radiologia medica. 2022. doi: 10.1007/s11547-022-01541-1 36018488

[31] Zamboglou C, Carles M, Fechter T, Kiefer S, Reichel K, Fassbender TF, et al. Radiomic features from PSMA PET for non-invasive intraprostatic tumor discrimination and characterization in patients with intermediate- and high-risk prostate cancer—a comparison study with histology reference. Theranostics. 2019;9:2595–605. doi: 10.7150/thno.32376 31131055 PMC6525993

[32] Buvat I, Orlhac F. The Dark Side of Radiomics: On the Paramount Importance of Publishing Negative Results. J Nucl Med. 2019;60:1543–4. doi: 10.2967/jnumed.119.235325 31541033

[33] Dissaux G, Visvikis D, Da-ano R, Pradier O, Chajon E, Barillot I, et al. Pretreatment ^18^F-FDG PET/CT Radiomics Predict Local Recurrence in Patients Treated with Stereotactic Body Radiotherapy for Early-Stage Non–Small Cell Lung Cancer: A Multicentric Study. Journal of Nuclear Medicine. 2020;61:814. doi: 10.2967/jnumed.119.228106 31732678

[34] Ferrández MC, Eertink JJ, Golla SSV, Wiegers SE, Zwezerijnen GJC, Pieplenbosch S, et al. Combatting the effect of image reconstruction settings on lymphoma [18F]FDG PET metabolic tumor volume assessment using various segmentation methods. EJNMMI Research. 2022;12:44. doi: 10.1186/s13550-022-00916-9 35904645 PMC9338209

[35] Hotta M, Minamimoto R, Gohda Y, Miwa K, Otani K, Kiyomatsu T, Yano H. Prognostic value of 18F-FDG PET/CT with texture analysis in patients with rectal cancer treated by surgery. Annals of Nuclear Medicine. 2021;35:843–52. doi: 10.1007/s12149-021-01622-7 33948903

[36] Kocak B, Durmaz ES, Ates E, Kilickesmez O. Radiomics with artificial intelligence: a practical guide for beginners. Diagn Interv Radiol. 2019;25:485–95. doi: 10.5152/dir.2019.19321 31650960 PMC6837295

