# Supplementary material for: Optimization and validation of 18F-DCFPyL PET radiomics-based machine learning models in intermediate- to high-risk primary prostate cancer
Source: PLoS One. 2023 Nov 9;18(11):e0293672. doi: 10.1371/journal.pone.0293672 (PMC10635444; doi:10.1371/journal.pone.0293672)
Supplement: S1 Table — AUC = area under the curve; RF = random forest; LR = logistic regression; LNI = lymph-node involvement; GS = Gleason score; ECE = extracapsular extension; SD = standard deviation; int. = internal; ext. = external; val. = validation; RFE = Recursive Feature Elimination; LASSO = Least Absolute Shrinkage and Selection Operator. *The number of radiomics features remaining after dimension reduction. (DOCX) [file pone.0293672.s001.docx]

**Table S1. The different AUCs for the prediction of lymph node involvement (LNI), extracapsular extension (ECE), and Gleason score (GS) per dataset with or without the addition of clinical features, omission of correlated radiomics features, and application of Combat harmonization.**

| **Outcome** | **Model** | **Add clinical** | **Drop correlated** | **Apply Combat** | **Training AUC** | **Int. val. AUC** | **Ext. val. AUC** | **No. of features*** | **Dimension reduction** |
| --- | --- | --- | --- | --- | --- | --- | --- | --- | --- |
| LNI | RF | False | False | False | 0.88 | 0.49 | 0.54 | 3 | LASSO |
| LNI | RF | False | False | True | - | - | 0.64 | 3 | LASSO |
| LNI | RF | False | True | False | 0.88 | 0.49 | 0.53 | 3 | LASSO |
| LNI | RF | False | True | True | - | - | 0.63 | 3 | LASSO |
| LNI | RF | True | False | False | 0.87 | 0.38 | 0.58 | 9 | LASSO |
| LNI | RF | True | False | True | - | - | 0.69 | 9 | LASSO |
| LNI | RF | True | True | False | 0.86 | 0.35 | 0.59 | 9 | LASSO |
| LNI | RF | True | True | True | - | - | 0.67 | 9 | LASSO |
| LNI | LR | False | False | False | 0.85 | 0.39 | 0.54 | 48 | Univariate |
| LNI | LR | False | False | True | - | - | 0.51 | 48 | Univariate |
| LNI | LR | False | True | False | 0.84 | 0.27 | 0.53 | 8 | Univariate |
| LNI | LR | False | True | True | - | - | 0.47 | 8 | Univariate |
| LNI | LR | True | False | False | 0.83 | 0.40 | 0.52 | 54 | Univariate |
| LNI | LR | True | False | True | - | - | 0.49 | 54 | Univariate |
| LNI | LR | True | True | False | 0.83 | 0.43 | 0.57 | 14 | Univariate |
| LNI | LR | True | True | True | - | - | 0.55 | 14 | Univariate |
| GS | RF | False | False | False | 0.84 | 0.78 | 0.76 | 128 | RFE |
| GS | RF | False | False | True | - | - | 0.74 | 128 | RFE |
| GS | RF | False | True | False | 0.82 | 0.85 | 0.66 | 20 | RFE |
| GS | RF | False | True | True | - | - | 0.74 | 20 | RFE |
| GS | RF | True | False | False | 0.87 | 0.76 | 0.71 | 134 | RFE |
| GS | RF | True | False | True | - | - | 0.73 | 134 | RFE |
| GS | RF | True | True | False | 0.89 | 0.87 | 0.74 | 26 | RFE |
| GS | RF | True | True | True | - | - | 0.79 | 26 | RFE |
| GS | LR | False | False | False | 0.84 | 0.93 | 0.61 | 3 | LASSO |
| GS | LR | False | False | True | - | - | 0.79 | 3 | LASSO |
| GS | LR | False | True | False | 0.83 | 0.89 | 0.60 | 3 | LASSO |
| GS | LR | False | True | True | - | - | 0.74 | 3 | LASSO |
| GS | LR | True | False | False | 0.88 | 0.94 | 0.59 | 9 | LASSO |
| GS | LR | True | False | True | - | - | 0.79 | 9 | LASSO |
| GS | LR | True | True | False | 0.88 | 0.92 | 0.58 | 9 | LASSO |
| GS | LR | True | True | True | - | - | 0.76 | 9 | LASSO |
| ECE | RF | False | False | False | 0.79 | 0.58 | 0.67 | 1 | LASSO |
| ECE | RF | False | False | True | - | - | 0.69 | 1 | LASSO |
| ECE | RF | False | True | False | 0.79 | 0.58 | 0.67 | 1 | LASSO |
| ECE | RF | False | True | True | - | - | 0.69 | 1 | LASSO |
| ECE | RF | True | False | False | 0.69 | 0.70 | 0.68 | 7 | LASSO |
| ECE | RF | True | False | True | - | - | 0.69 | 7 | LASSO |
| ECE | RF | True | True | False | 0.68 | 0.70 | 0.68 | 7 | LASSO |
| ECE | RF | True | True | True | - | - | 0.69 | 7 | LASSO |
| ECE | LR | False | False | False | 0.78 | 0.57 | 0.63 | 1 | LASSO |
| ECE | LR | False | False | True | - | - | 0.63 | 1 | LASSO |
| ECE | LR | False | True | False | 0.78 | 0.57 | 0.63 | 1 | LASSO |
| ECE | LR | False | True | True | - | - | 0.63 | 1 | LASSO |
| ECE | LR | True | False | False | 0.75 | 0.58 | 0.63 | 7 | LASSO |
| ECE | LR | True | False | True | - | - | 0.63 | 7 | LASSO |
| ECE | LR | True | True | False | 0.76 | 0.58 | 0.63 | 7 | LASSO |
| ECE | LR | True | True | True | - | - | 0.63 | 7 | LASSO |

AUC = area under the curve; RF = random forest; LR = logistic regression; LNI = lymph-node involvement; GS = Gleason score; ECE = extracapsular extension; SD = standard deviation; int. = internal; ext. = external; val. = validation; RFE = Recursive Feature Elimination; LASSO = Least Absolute Shrinkage and Selection Operator.
*The number of radiomics features remaining after dimension reduction
