# Supplementary material for: Optimization and validation of 18F-DCFPyL PET radiomics-based machine learning models in intermediate- to high-risk primary prostate cancer
Source: PLoS One. 2023 Nov 9;18(11):e0293672. doi: 10.1371/journal.pone.0293672 (PMC10635444; doi:10.1371/journal.pone.0293672)
Supplement: S2 Table — Derived from a Random Forest and Logistic Regression using different machine-learning configurations (Table 1). (DOCX) [file pone.0293672.s002.docx]

**Table S2. Selected features after dimension reduction for the prediction of lymph node involvement (LNI), extracapsular extension (ECE), and Gleason score (GS).**Derived from a Random Forest and Logistic Regression using different machine-learning configurations (Table 1).

| **LNI - RF** | **LNI - LR** | **ECE - RF** | **ECE - LR** | **GS - RF** | **GS - LR** |
| --- | --- | --- | --- | --- | --- |
| Least axis length | Approximate volume | Grey level non uniformity GLSZM.2 | Grey level non uniformity GLSZM.2 | Volume | Inverse difference moment normalised.2 |
| Joint entropy | Surface |  |  | Approximate volume | Zone distance non uniformity GLDZM.1 |
| Cluster shade.2 | Least axis length | |  | Surface | Zone distance non uniformity GLDZM.2 |
|  | Integrated intensity | |  | Spherical disproportion | |
|  | Energy |  |  | Sphericity |  |
|  | Volume at int fraction 10 | |  | Center of mass shift | |
|  | Difference vol at int fraction | |  | Maximum 3D diameter | |
|  | Energy.1 |  |  | Major axis length | |
|  | Joint maximum | |  | Least axis length | |
|  | Joint entropy | |  | Elongation |  |
|  | Sum entropy |  |  | Vol density AABB | |
|  | Angular second moment | |  | Integrated intensity | |
|  | Correlation |  |  | Morans I |  |
|  | Cluster shade | |  | Local intensity peak | |
|  | Joint entropy.2 | |  | Kurtosis |  |
|  | Correlation.2 |  |  | Quartile coefficient | |
|  | Cluster shade.2 | |  | Volume at int fraction 90 | |
|  | Inverse difference moment normalised.4 | |  | Difference vol at int fraction | |
|  | Inverse difference moment normalised.5 | |  | Skewness.1 |  |
|  | Run length non uniformity | |  | Kurtosis.1 |  |
|  | Run entropy |  |  | Quartile coefficient.1 | |
|  | Run length non uniformity.1 | |  | Energy.1 |  |
|  | Run length non uniformity.2 | |  | Maximum histogram gradient | |
|  | Run entropy.2 | |  | Joint maximum | |
|  | Run length non uniformity.3 | |  | Joint entropy |  |
|  | Run length non uniformity.4 | |  | Difference entropy | |
|  | Run length non uniformity.5 | |  | Sum entropy |  |
|  | Zone size non uniformity | |  | Angular second moment | |
|  | Zone size entropy | |  | Inverse difference normalised | |
|  | Zone size non uniformity.1 | |  | Inverse difference moment | |
|  | Coarseness.1 |  |  | Inverse difference moment normalised | |
|  | Coarseness.2 |  |  | Cluster shade |  |
|  | Large distance emphasis gldzm | |  | Second measure of information correlation | |
|  | Zone distance non uniformity gldzm | |  | Joint maximum.1 | |
|  | Zone distance non uniformity normalized gldzm | | | Joint entropy.1 | |
|  | Zone distance variance gldzm | |  | Angular second moment.1 | |
|  | Zone distance entropy gldzm | |  | Inverse difference normalised.1 | |
|  | Large distance emphasis gldzm.1 | |  | Inverse difference moment normalised.1 | |
|  | Zone distance non uniformity gldzm.1 | |  | Inverse variance.1 | |
|  | Zone distance non uniformity normalized gldzm.1 | | | Correlation.1 |  |
|  | Zone distance variance gldzm.1 | |  | Joint maximum.2 | |
|  | Zone distance non uniformity gldzm.2 | |  | Joint entropy.2 | |
|  | Dependence count non uniformity | |  | Sum entropy.2 |  |
|  | Dependence count entropy | |  | Angular second moment.2 | |
|  | Dependence count energy | |  | Inverse difference normalised.2 | |
|  | Dependence count non uniformity.1 | |  | Inverse difference moment normalised.2 | |
|  | Dependence count non uniformity.2 | |  | Inverse variance.2 | |
|  | Dependence count entropy.2 | |  | Cluster shade.2 | |
|  |  |  |  | First measure of information correlation.2 | |
|  |  |  |  | Second measure of information correlation.2 | |
|  |  |  |  | Joint maximum.3 | |
|  |  |  |  | Joint entropy.3 | |
|  |  |  |  | Inverse difference normalised.3 | |
|  |  |  |  | Inverse difference moment normalised.3 | |
|  |  |  |  | Correlation.3 |  |
|  |  |  |  | First measure of information correlation.3 | |
|  |  |  |  | Second measure of information correlation.3 | |
|  |  |  |  | Joint entropy.4 | |
|  |  |  |  | Angular second moment.4 | |
|  |  |  |  | Inverse difference normalised.4 | |
|  |  |  |  | Inverse difference moment normalised.4 | |
|  |  |  |  | Inverse difference normalised.5 | |
|  |  |  |  | Inverse difference moment normalised.5 | |
|  |  |  |  | Correlation.5 |  |
|  |  |  |  | First measure of information correlation.5 | |
|  |  |  |  | Short run emphasis | |
|  |  |  |  | Long runs emphasis | |
|  |  |  |  | Short run low grey level emphasis | |
|  |  |  |  | Grey level non uniformity normalized | |
|  |  |  |  | Run length non uniformity | |
|  |  |  |  | Run length variance | |
|  |  |  |  | Run entropy |  |
|  |  |  |  | Long runs emphasis.1 | |
|  |  |  |  | Run length non uniformity.1 | |
|  |  |  |  | Run percentage.1 | |
|  |  |  |  | Long runs emphasis.2 | |
|  |  |  |  | Short run low grey level emphasis.2 | |
|  |  |  |  | Grey level non uniformity normalized.2 | |
|  |  |  |  | Run length non uniformity.2 | |
|  |  |  |  | Run entropy.2 |  |
|  |  |  |  | Run length non uniformity.3 | |
|  |  |  |  | Grey level non uniformity.4 | |
|  |  |  |  | Run length non uniformity.4 | |
|  |  |  |  | Run length non uniformity.5 | |
|  |  |  |  | Large zone high grey level emphasis | |
|  |  |  |  | Grey level non uniformity GLSZM | |
|  |  |  |  | Grey level non uniformity normalized GLSZM | |
|  |  |  |  | Zone size entropy | |
|  |  |  |  | Small zone emphasis.1 | |
|  |  |  |  | Large zone emphasis.1 | |
|  |  |  |  | Large zone high grey level emphasis.1 | |
|  |  |  |  | Zone size non uniformity.1 | |
|  |  |  |  | Grey level non uniformity GLSZM.2 | |
|  |  |  |  | Zone size non uniformity.2 | |
|  |  |  |  | Zone size entropy.2 | |
|  |  |  |  | Coarseness |  |
|  |  |  |  | Contrast.6 |  |
|  |  |  |  | Busyness |  |
|  |  |  |  | Coarseness.1 |  |
|  |  |  |  | Coarseness.2 |  |
|  |  |  |  | Small distance emphasis GLDZM | |
|  |  |  |  | Large distance emphasis GLDZM | |
|  |  |  |  | Small distance low grey level emphasis GLDZM | |
|  |  |  |  | Grey level non uniformity GLDZM | |
|  |  |  |  | Grey level non uniformity normalized GLDZM | |
|  |  |  |  | Zone distance non uniformity GLDZM | |
|  |  |  |  | Zone distance non uniformity normalized GLDZM | |
|  |  |  |  | Grey level variance GLDZM | |
|  |  |  |  | Zone distance variance GLDZM | |
|  |  |  |  | Zone distance entropy GLDZM | |
|  |  |  |  | Zone distance non uniformity GLDZM.1 | |
|  |  |  |  | Grey level non uniformity GLDZM.2 | |
|  |  |  |  | Zone distance non uniformity GLDZM.2 | |
|  |  |  |  | Zone distance non uniformity normalized GLDZM.2 | |
|  |  |  |  | Zone distance variance GLDZM.2 | |
|  |  |  |  | Low dependence emphasis | |
|  |  |  |  | Grey level non uniformity normalized.6 | |
|  |  |  |  | Dependence count non uniformity | |
|  |  |  |  | Dependence count non uniformity normalized | |
|  |  |  |  | Dependence count entropy | |
|  |  |  |  | Dependence Count Energy | |
|  |  |  |  | High dependence high grey level emphasis.1 | |
|  |  |  |  | Dependence count non uniformity.1 | |
|  |  |  |  | Low dependence low grey level emphasis.2 | |
|  |  |  |  | High dependence high grey level emphasis.2 | |
|  |  |  |  | Dependence count non uniformity.2 | |
|  |  |  |  | Dependence count entropy.2 | |
|  |  |  |  | Dependence Count Energy.2 | |
